# Multiple Imaging and Surgical Characteristics in Cardiac Metastasis from Undifferentiated Uterine Sarcoma

**DOI:** 10.1155/2022/6025354

**Published:** 2022-11-11

**Authors:** Hiroka Sasahara, Shoichiro Yatsu, Hideki Wada, Eiryu Sai, Soshi Moriya, Suguru Miyazaki, Kan Kajimoto, Manabu Ogita, Satoru Suwa

**Affiliations:** ^1^Department of Cardiovascular Medicine, Juntendo University Shizuoka Hospital, Izunokuni-shi, Japan; ^2^Department of Metabolism and Endocrinology, Juntendo University Graduate School of Medicine, Tokyo, Japan; ^3^Department of Cardiovascular Biology and Medicine, Juntendo University Graduate School of Medicine, Tokyo, Japan; ^4^Cardiovascular Surgery, Juntendo University Shizuoka Hospital, Izunokuni-shi, Japan

## Abstract

Although cardiac metastasis of malignant tumors has often been reported, undifferentiated uterine sarcoma (UUS) is a rare and aggressive uterine tumor. Thus, little is known of the UUS as a primary site of cardiac metastasis. We report a case of a 66-year-old woman, with a history of uterine myoma for 30 years, who was hospitalized with a large uterine tumor and cardiac masses. Although we investigated cardiac masses using imaging modalities, such as ultrasound, cardiac computer tomography, and magnetic resonance imaging, it was challenging to determine the masses as metastasis or thrombi. Cardiac masses were removed by surgery to assess the tissue characteristics and were later identified as tumors due to their appearance. Then, pathological findings revealed that UUS spreads to the right ventricle. We attempted chemotherapy after surgery; however, the disease progressed very quickly and the patient died on the 49th day of admission. In this report, we described the case of a patient with a difficult diagnosis and rapid disease progression of cardiac metastasis from UUS.

## 1. Introduction

The incidence of cardiac metastases has increased over the last 30 years due to the increased life expectancy in oncologic patients owing to the advanced cancer diagnosis and management [1]. Uterine sarcomas are rare malignant tumors arising from the mesenchymal tissues of the uterus, i.e., the endometrial stroma, uterine muscle, and connective tissue [2]. Although it is estimated that 0.1–0.3% of the patients operated on for presumed uterine myoma have a uterine sarcoma, uterine sarcomas only represent 3–7% of all uterine malignances [3]. Thus, uterine sarcomas are relatively rare as the primary lesion of cardiac metastasis [1].

Undifferentiated uterine sarcoma (UUS) is one of the uterine sarcomas with a very poor prognosis, and most of the patients with UUS die within 2 years of diagnosis [2]. In addition, UUS is one of the rarest of the disease, with a prevalence of less than 10% among all uterine sarcomas [2]. Thus, due to its infrequent occurrence and adverse outcome, cardiac metastasis from UUS has never been reported. Although one report suggested the utility of ultrasound to differentiate the uterine sarcomas [4], approaches to UUS either in diagnosis or systemic therapy remain controversial despite the necessity for early diagnosis to treat this rapidly progressive disease. Herein, we presented a case of right ventricle metastases from UUS to report the imaging characteristics and state of rapid progress.

## 2. Case Report

**Figure 1:**
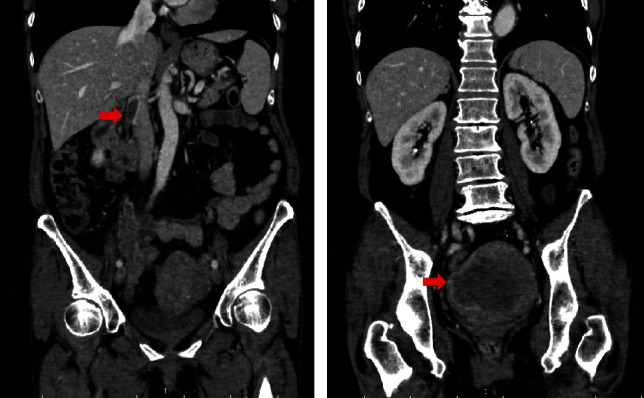
Computed tomography on admission. Intraluminal filling defect from the right ovarian vein to the inferior vena cava (arrow) (a) and a large uterine mass (arrow) (b).

A 66-year-old woman experienced abdominal pain and visited a private medical center. The patient had had a medical history of uterine myoma for 30 years, but no other history was reported. Computed tomography (CT) showed enlarged uterine myoma and a right ventricular mass and she was immediately transferred to our hospital. She had a blood pressure of 132/68 mmHg, a heart rate of 78 b.p.m., respiratory rate of 18 breaths/min, and oxygen saturation of 96% on room air at arrival. On initial physical examination, she had regular cardiac rhythm with no heart murmurs and no significant pretibial edema. Chest radiography showed neither congestion nor infiltrative shadows. Electrocardiography showed normal sinus rhythm without any ST segment change. Blood tests indicated a hemoglobin level of 10.7 g/L, C-reactive protein level of 1.10 mg/dL, and a D-dimer level of 8.3 *μ*g/mL. Tumor markers were within normal limits, including CA125 of 8 U/mL. A transthoracic echocardiogram showed an elongated mass-like structure in the right ventricle without any other complications. Transabdominal and transvaginal ultrasound showed multiple uterine solid masses (60 mm) with well-defined margins and mild extracellular fluid space. Contrast-enhanced CT revealed a very large uterine mass (107.5 mm), intraluminal filling defect via the right ovarian vein to the inferior vena cava (Figure 1), and right ventricle masses.

Experienced gynecologists and radiologists discussed and evaluated the CT scan findings. Although the uterine mass seemed to be a malignant tumor based on its configuration, tissue diagnosis was necessary to choose the adequate treatment. Multiple transvaginal biopsies were performed, but a diagnosis of normal uterine tissue was reached by a dedicated pathologist. However, diagnosing uterine mass with transvaginal biopsy has a low accuracy. Surgical mass resection was required, but the presence of cardiac masses made a surgical approach for uterine mass difficult due to the risk of embolization.

**Figure 2:**
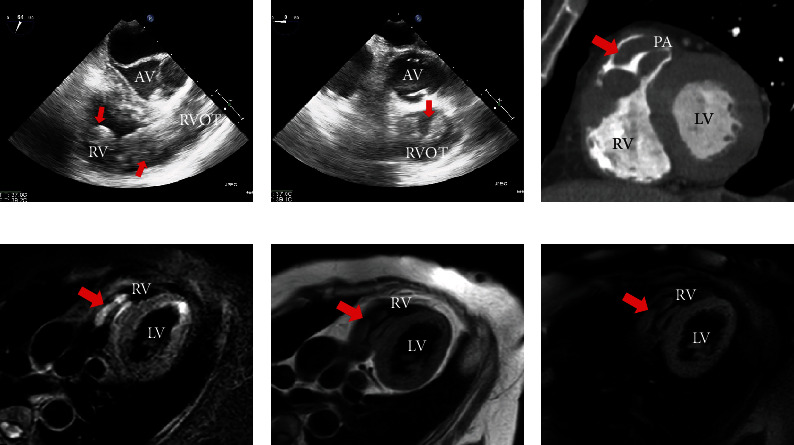
Transesophageal echocardiography, cardiac CT, and MRI appearance. Transesophageal echocardiography showed elongated mass-like structure (arrow) in the right ventricle (a, b). Short axis view of cardiac computed tomography scan revealed a well-defined mass lacking contrast enhancement in the right ventricle below the pulmonary artery valve (c), and cardiac magnetic resonance imaging showed some masses which had hypersignal intensity on fat-saturated T2-weighted image (d), isointensity on T1-weighted image (e), and no suppressed signal on fat-saturated T1-weighted image (f). (AV: atrioventricular; RV: right ventricle; RVOT: right ventricular outflow tract; LV: left ventricle; RV: right ventricle).

Thus, cardiologists were consulted and conducted further assessments for the cardiac mass. Transesophageal echocardiography revealed a well-defined, mobile mass below the pulmonary artery valve (Figures 2(a) and 2(b)). The cardiac CT scan and cardiac magnetic resonance imaging (MRI) were performed by experienced cardiologists and radiologists to assess further the anatomical and qualitative details. The cardiac CT revealed a well-defined mass with a lack of contrast enhancement in the right ventricle below the pulmonary artery valve (Figure 2(c)). The cardiac MRI showed some masses in the right ventricle, which had hypersignal intensity on fat-saturated T2-weighted image (Figure 2(d)), isointensity on T1-weighted image (Figure 2(e)), and no suppressed signal by fat suppression on T1-weighted image (Figure 2(f)). Based on these images, it was difficult to identify them as either tumors or thrombi. In addition, the mass did not respond well to anticoagulants, with its size remaining unchanged after one week of rivaroxaban (15 mg twice daily: Japanese standard dose for venous thromboembolism).

**Figure 3:**
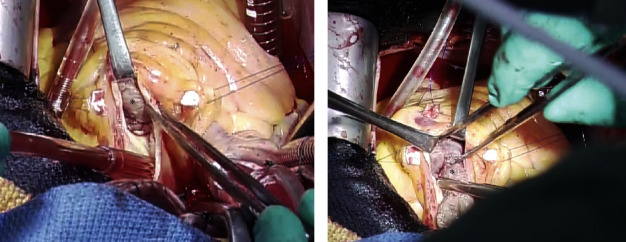
Surgical images via pulmonary artery incision. A large mass (asterisk) in the main pulmonary artery and in the right ventricle via the pulmonary artery incision (a) was removed from the right ventricle (b).

Our cardiologists and gynecologists repeatedly discussed the approach on this case and decided to perform an open-heart mass removal for tissue diagnosis and to remove the embolic source. On the 20th day of hospitalization, cardiac surgery was performed with median sternotomy by making an incision along the right atrium and main pulmonary artery. A large mass was observed in the main pulmonary artery and right ventricle. The mass was identified as a tumor due to a solid and elastic appearance. It perforated the pulmonary valve and infiltrated the right ventricular wall (Figure 3). Intraoperative rapid pathological findings showed hemorrhagic necrosis, interstitial edema, and dense growth of anaplastic cells, indicating cardiac metastasis. At this stage, we had done only tumor resection and closed her chest. Moreover, immunostaining showed diffusely positive staining for vimentin, partially positive for CD10, CD68, and Ki-67, and negative results for desmin, CD34, factor VIII, and estrogen receptor, which led to the diagnosis of undifferentiated sarcoma. These results suggest that UUS spreads to the right ventricle via the right ovarian vein and inferior vena cava.

The patient's postoperative recovery was uneventful and she decided to undergo chemotherapy. However, she experienced severe nausea and dyspnea on the 15th day after surgery. Contrast-enhanced CT revealed recurrence of the right ventricle mass, pulmonary embolism, intestinal obstruction, and multiple pulmonary metastases. The patient died 29 days postsurgery.

## 3. Discussion

This case presents a rapidly deteriorating condition with difficulty in diagnosing cardiac metastasis from UUS, which was pathologically diagnosed after open cardiac surgery. Cardiac metastases of malignant tumors are relatively common, ranging from 0.7% to 3.5% in autopsy cases, despite low diagnosis rates in individuals during their lives [1]. Although cardiac metastasis is highly reported in lung cancer, breast cancer, leukemia, malignant lymphoma, and malignant melanoma, only few cases have been reported on metastasis in uterine sarcoma [5, 6]. While imaging studies are important, the usage of these modalities in diagnosing cardiac mass has not been determined. Echocardiography is the first choice for assessing cardiac masses and it verifies their location, size, and mobility [7, 8]. Also, cardiac CT reveals anatomical construction with excellent spatial resolution whereas MRI offers excellent contrast resolution and unparalleled, direct multiplanar assessment. However, the low incidence and diversity of cardiac mass make it difficult to identify the mass and diagnose the condition using only imaging modalities [9, 10]. Positron emission tomography (PET) CT is known as the useful diagnostic tool for the malignant disease; however, it is not suitable to discriminate uterine myoma and sarcoma and it is difficult to judge cardiac malignant tumors because of the physiological accumulation in the heart. To our knowledge, this is the first case report that describes the characteristics of cardiac CT and MRI of metastasis from UUS. In this case, although high T2- and isointensity T1-weighted signals were indicative of metastatic tumors, thrombus could not be ruled out due to the characteristics of clots in MRI findings which gradually changed according to the duration from the onset of thrombus [9]. Therefore, improving the accuracy of the imaging modalities of cardiac metastasis from UUS is required to manage patients with cardiac masses.

Uterine sarcomas are rare tumors that constitute 3%–7% of uterine neoplasms [3]. They are heterogeneous pathological neoplasms characterized by a more aggressive and poorer prognosis than endometrial cancer. Endometrial stromal sarcoma (ESS) is a uterine mesenchymal tumor that can be categorized into four categories according to the 2014 World Health Organization classification: endometrial stromal nodule, low-grade ESS, high-grade ESS, and UUS [2]. UUS is classified as a high-grade sarcoma and is one of the most aggressive uterine tumors that typically arises in the endometrium and is cytologically atypical and highly mitotic. UUS usually develops in postmenopausal women with common manifestations including a pelvic mass and abnormal vaginal bleeding. Patients with UUS are usually present with extrauterine involvement at the time of diagnosis and are quite advanced [2]. Although total hysterectomy, with or without lymphadenectomy and bilateral oophorectomy, is used in the management of UUS, nonsurgical interventions for UUS are still under investigation because of lack of accurate evidence regarding UUS sensitivity to radiation and chemotherapy [3].

In the present case, the preoperative diagnosis of endocardial and uterine masses was difficult and open-heart surgery led to the diagnosis of a metastatic cardiac tumor and UUS. Due to the highly invasive nature of cardiac surgery, a nonsurgical approach is required for the diagnosis of cardiac mass. UUS is a very rare disease with rapid progression and therefore, early diagnosis and therapeutic intervention are necessary. To the best of our knowledge, this is the first to report the case of cardiac metastasis from UUS. In the future, accumulating further similar cases is important to discuss the diagnostic and therapeutic processes of cardiac metastasis from UUS.

## Data Availability

Data sharing is not applicable to this article as no datasets were generated or analyzed during the current study.
